# Extrinsic Factors Regulating Dendritic Patterning

**DOI:** 10.3389/fncel.2020.622808

**Published:** 2021-01-13

**Authors:** Tzu-Yang Lin, Pei-Ju Chen, Hung-Hsiang Yu, Chao-Ping Hsu, Chi-Hon Lee

**Affiliations:** ^1^Institute of Cellular and Organismic Biology, Academia Sinica, Taipei, Taiwan; ^2^Institute of Chemistry, Academia Sinica, Taipei, Taiwan

**Keywords:** dendritic development, ligand-receptor, glomerular targeting, layer-specific targeting, dendritic tiling, dendritic field size, intercellular communication

## Abstract

Stereotypic dendrite arborizations are key morphological features of neuronal identity, as the size, shape and location of dendritic trees determine the synaptic input fields and how information is integrated within developed neural circuits. In this review, we focus on the actions of extrinsic intercellular communication factors and their effects on intrinsic developmental processes that lead to dendrite patterning. Surrounding neurons or supporting cells express adhesion receptors and secreted proteins that respectively, act *via* direct contact or over short distances to shape, size, and localize dendrites during specific developmental stages. The different ligand-receptor interactions and downstream signaling events appear to direct dendrite morphogenesis by converging on two categorical mechanisms: local cytoskeletal and adhesion modulation and global transcriptional regulation of key dendritic growth components, such as lipid synthesis enzymes. Recent work has begun to uncover how the coordinated signaling of multiple extrinsic factors promotes complexity in dendritic trees and ensures robust dendritic patterning.

## Introduction: Dendritic Forms Follow Functions

Neurons form complex yet stereotyped branching dendritic arbors, which receive and process information from other neurons. The locations of dendritic arbors determine the types of presynaptic partners and input information that is received and integrated, while the dendritic shape, size and complexity govern the input number and passive electrotonic properties (London and Hausser, 2005; Lefebvre et al., 2015). Stereotypical dendrite arborizations are tightly correlated with neuronal identity and functions. Quantitative analyses of pyramidal and Purkinje cells suggest that their dendritic morphology maximizes the complexity of potential inputs under the constrain of total dendritic lengths while theoretical modeling of neocortical neurons suggests that changes in dendritic morphology are able to alter signal propagation within the neuron (Mainen and Sejnowski, 1996; Wen et al., 2009). Thus, dendrite shapes and sizes can conceivably affect synaptic connectivity and neuronal computation. Moreover, failures to establish proper dendritic structures have been observed in human pathological studies of neurological and neurodevelopmental disorders (Kulkarni and Firestein, 2012; Forrest et al., 2018).

During brain development, each neuron runs a temporal cell-intrinsic growth program and also responds to dynamic environmental cues, with interplay between these extrinsic factors and intrinsic processes ensuring proper dendritic morphogenesis. Dendrite development requires specific intrinsic factors, such as transcriptional regulators, that facilitate growth of neurons and allow the cells to acquire subtype-specific morphologies (Jan and Jan, 2010; Dong et al., 2015). Additionally, recent genetic and transcriptomic analyses have revealed that different types of neurons express distinct cell surface proteins that respond to external cues in order to guide and shape dendrites (Li et al., 2017; Kurmangaliyev et al., 2019; Davis et al., 2020; Jain et al., 2020). This review focuses on the morphological aspects instructed by secreted and contact-mediated factors and the mechanisms by which extrinsic cues and key intrinsic regulators are spatiotemporally coordinated to shape dendritic patterning. First, we describe current work on different neural architectures, highlighting notable aspects of dendritic routing related to each architecture. We then summarize the recently uncovered mechanisms of action that mediate dendritic patterning in response to extrinsic factors at various dendritic developmental stages. Finally, we discuss the coordination of multiple extrinsic factors in regulating dendritic development.

## Dendritic Patterning in Different Neural Architectures

**FIGURE 1:**
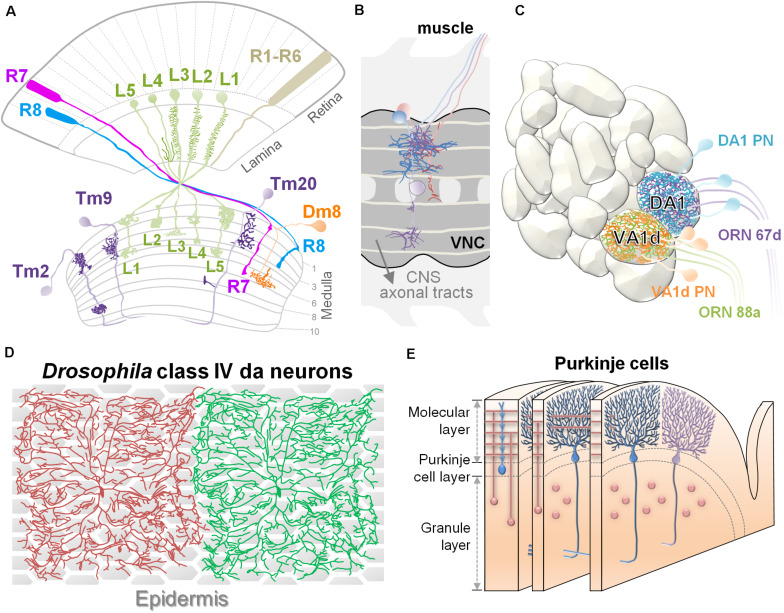
Experimental systems for studying dendritic patterning. **(A)** Organization of layers and columns in the *Drosophila* visual system. Schematic illustration shows the structures of retina, lamina, and medulla. Dendrites of lamina neurons (L1∼L5 in green) received visual information from photoreceptors and organized in a columnar structure. Transmedulla neurons (Tm2, 9, and 20 in purple) elaborate their dendrites into specific layers and are confined to a single medulla column. The amacrine-like neuron Dm8 (orange) extends dendrites in the M6 layer where they receive ∼14 R7 inputs. **(B)** The illustration depicts *Drosophila* embryonic abdomen motoneurons that project their dendritic arbors within the ventral nerve cord of the embryonic CNS in partial segments. The aCC neuron is magenta. FasII-positive longitudinal axon bundles are light yellow. CNS axonal tracts are labeled in gray. Anterior is to the left. **(C)** Anatomical organization of the *Drosophila* olfactory system. The antennal lobe is organized into discrete neuropil compartments, called glomeruli, where matched axonal arbors of olfactory receptor neurons (ORNs) and dendrites of projection neurons (PNs) are converged precisely. This drawing shows two adjacent glomeruli located at the dorsolateral region of the AL, the Or67d:DA1 and Or88a:VA1d. Specific types of projection neurons (PNs) project their dendrites to discrete glomeruli within the antennal lobe. In panels **(A–C)**, dendritic arbors are highlighted in dark color. **(D)** In third-instar *Drosophila* larva, the dendrites of a highly branched class IV dendritic arborization (C4da) neurons achieve almost complete coverage of the body wall. Dendrites from the same cell or from the same class of neurons do not overlap in their territories. Epidermis is shown as hexagon shapes underneath the C4da neurons. **(E)** The schematic shows the general organization of Purkinje cells and granule cells in the cerebellar cortex. Elaborate dendritic trees of adjacent Purkinje cells lie parallel in planes and form synapses with T-shaped parallel fibers (in pink), the axons of granule cells.

Recent studies using genetics and imaging analysis have greatly advanced the identification of extrinsic factors and their roles in dendritic morphogenesis. These studies have focused on multiple experimental systems with unique neural architectures, such as *Drosophila* adult visual neurons (Fischbach and Dittrich, 1989; Ting et al., 2014), *Drosophila* larval dendritic arborization (da) sensory neurons (Jan and Jan, 2010), *C. elegans* PVD neurons (Inberg et al., 2019), mouse retinal neurons (Sanes and Zipursky, 2010), pyramidal neurons (Spruston, 2008), and cerebellar Purkinje cells (Fujishima et al., 2018; Figure 1). Different types of neural architectures have distinct requirements for dendritic routing, and consequently, the sources and patterns of extrinsic factors that guide routing differ between the model systems. Three major types of neural architectures have been examined in detail, including layer-column, glomeruli, and 2D-space tiling.

### Routing Dendrites in Layers and Columns

**FIGURE 2:**
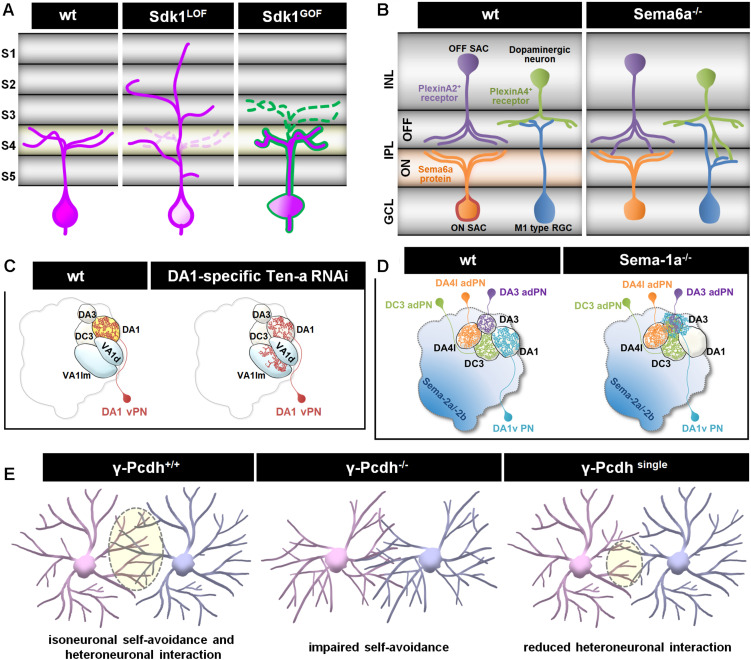
Different cellular mechanisms regulate dendritic patterning. **(A)** Sidekick 1 (Sdk1) transmembrane adhesion receptor is concentrated in a distinct set of IPL sublaminae (S4 and light orange) in vertebrate retina sections. Sdk molecules can bind homophilically and extend dendritic arbors to one or a few restricted IPL sublaminae. The absence of Sdk1 causes dendrite mistargeting from S4 to other layers. Ectopically expressed Sdk1 (magenta) in Sdk1-non-expressing cells (outlined in green) redirects dendrites to the S4 layer. Either loss-of-function or gain-of-function for Sdks results in the degradation of cell-type-specific laminar restriction and leads to impaired motion sensing, due to selective loss of specific synapses (Yamagata and Sanes, 2008, 2018; Krishnaswamy et al., 2015). **(B)** Semaphorins and plexins function as repellent cues in control of dendrite targeting (Koropouli and Kolodkin, 2014). Transmembrane Sema6A is selectively expressed by RGCs and amacrine cells in most ON sublaminae; its receptor, PlexinA2 or PlexinA4, is expressed complementarily in OFF sublaminae of the IPL in the developing mouse retina. In ligand or receptor mutants, PlexinA2+ or PlexinA4 + amacrine cell dendrites are misrouted to abnormal locations in the ON IPL (Matsuoka et al., 2011a,b; Sun et al., 2013). INL, Inner nuclear layer; IPL, Inner plexiform layer; GCL, Ganglion cell layer. **(C)** Two *Drosophila* Teneurins, Ten-a and Ten-m, exhibit complementary expression patterns in the AL. Epidermal growth factor-repeat containing transmembrane Tens bind homophilically and act as attractive cues to recruit the relevant synaptic partners. Reduce expression of Ten-a in PNs redirects partial of their dendrites to glomeruli where presynaptic afferents express low Ten-a levels (Hong et al., 2012). **(D)** Repulsive transmembrane protein Semaphorin-1a (Sema-1a) regulates appropriate PN dendritic targeting to destined glomeruli in the AL. Dendrites of Sema-1a-difficient PNs mistarget and/or innervate into the DA3 glomerulus (Shen et al., 2017). **(E)** A family of molecular diversity cell recognition molecules, Pcdhs, is required to mediate dendrite self-avoidance and heteroneuronal interaction during development. Similar to *Drosophila* Dscam1, the Pcdhs are required for self-avoidance, with an analogous role for self/non-self-discrimination in mouse retinal starburst amacrine cells (SACs) and cerebellar Purkinje cells. Expressing a single γ-Pcdh isoform in γ-Pcdh-knockout is sufficient to rescue self-avoidance but reduces heteroneuronal dendrite interactions in SACs (Lefebvre et al., 2012).

In the visual systems of vertebrates and invertebrates, neurons extend dendrites to particular layers of the stratified neuropil in the retina and brain (Sanes and Zipursky, 2010). In the vertebrate retina, the laminar arrangement of visual neurons is separated into three distinct “nuclear” layers (contain cell bodies but no synapses) interspersed with two “plexiform” layers (contain synapses but no cell bodies). Axons of ON bipolar cells (excited by light) terminate in the inner half of the inner plexiform layer (IPL), where they form synapses with dendrites of ON retinal ganglion cells (RGCs) and amacrine cells. Similarly, OFF bipolar axons and OFF RGCs dendrites form synapses in the outer half of the IPL. The RGC axons relay visual information and innervate the optic tectum (also called the superior colliculus), which is also composed of stacked layers that each encode certain visual features, such as light polarity or direction-specific motion (Sanes and Zipursky, 2010; Figures 2A,B). Similar stratified neuropils are also found in invertebrate visual systems. In the *Drosophila* optic lobe, the majority of dendritic branches arise from one or two nodes in specific layers, with the dendrites extending to different layers. For example, Tm20 neurons extend most dendrites from the third medulla layer (M3) to the M1–M3 layers, while Dm8 neurons extend most of their dendrites in the M6 layer (Fischbach and Dittrich, 1989; Ting et al., 2014; Figure 1A). In addition to layer-specific targeting, dendrites from medulla neurons also exhibit type-specific planar directions of projection. For example, Tm1, Tm2, and Tm9 neurons extend dendrites anteriorly, while Tm20 neurons project dendrites posteriorly (Ting et al., 2014; Figure 1A). The development of this grid-like organization of the visual systems requires matching axonal terminals and dendrites in layers and controlling dendritic elaboration in columns. The extrinsic factors that regulate dendritic development are often provided by the grid-forming afferents. Surface receptors serve as adhesive or repulsive cues to regulate layer-specific elaboration of dendrites. The secreted factors often act in short-range to pattern dendrites and to control the field sizes (Figures 2A,B; detail molecular signals will be discussed in the below sections).

*Drosophila* embryonic abdomen motorneurons are organized in an analogous grid-like organization. A set of ∼80 motoneurons are present in each segment of the ventral nerve cord (VNC) (Figure 1B), and each motorneuron projects an axon along a distinct nerve to innervate a peripheral target muscle field with characteristic dendritic arborization (Landgraf et al., 1997). The segmental muscular and longitudinal neuronal structures serve as landmarks for dendrite morphological analysis. For instance, developing aCC (anterior corner cell) motor neurons (magenta cell in Figure 1B) can be easily located and manipulated for studies on the dynamics of dendritic arbor growth (Tripodi et al., 2008). In addition, stereotypical dendritogenesis sites on aCC neurons are well suited for investigating the molecular mechanisms that control selection of dendritic branch points (Kamiyama et al., 2015). Using the well-aligned and organized reference architectures in the above mentioned systems, one can effectively quantify several different aspects of morphological alterations, including dendritic initiation, branching, and termination.

### Glomerular Targeting

In the *Drosophila* olfactory system, odorant neurons (ORNs) relay odorant information to the primary olfactory center (specialized neuropil structures called glomeruli), in the antennal lobe/olfactory bulb. Specific types of insect olfactory projection neurons (PNs) and vertebrate mitral cells precisely target complex dendrites to discrete glomeruli (Figures 1C, 2C,D), where they receive olfactory information from specific ORNs. The axons of these neurons then project to higher brain processing centers. This system is highly amenable to studies on dendritic guidance and targeting mechanisms. Previous studies have shown that glomerular targeting of PN dendrites is controlled by intrinsic factors related to cell lineage and identity and further regulated by extrinsic cues (Corty et al., 2009).

Dendritic targeting to discrete glomeruli is likely achieved by a combination of two mechanisms: gradients of diffusible morphogens might act in long-range to pattern glomeruli while contact-dependent adhesive or repulsive cues match PN dendrites to ORN axons. During development, multiple ligands are secreted by ORNs and form gradients along dorsolateral-ventromedial (DL-VM) axis in the antennal lobe (Figure 2D; Sweeney et al., 2011; Wu et al., 2014; Hing et al., 2020) while different types of PN dendrites expressing distinct levels of receptors to generate quantitative signaling to orient PN dendritic innervations. In addition, class-specific surface proteins or receptors potentially refine specific glomerular targeting locally *via* short-range contact-mediated action (Figure 2C; Hong et al., 2009, 2012; Ward et al., 2015; Xie et al., 2019). Collectedly, pre-defined molecular gradients and local interactions suggest a combinatorial molecular code allowing the precise targeting of diverse neuron types within the antennal lobe.

### Tiling on a Two-Dimensional Space

The most striking and characteristic features of the polymodal sensory da (dendritic arborization) neurons in *Drosophila* and the cerebellar Purkinje cells in vertebrates are their large and highly branched dendritic patterns on a 2D space. Despite their considerable sizes and complexities, the dendritic arbors originating from the same cell or different cells, do not cross, fasciculate or entangle, but together, the arbors maximize coverage of the 2D space (Figures 1D,E). A prerequisite to achieve 2D tiling of dendrites is to restrict dendritic growth on a 2D surface where contact-dependent repulsion among dendrites can exert its effects (Han et al., 2012).

Two related processes that both utilize contact-dependent repulsion are self-avoidance and tiling (Zipursky and Grueber, 2013; Parrish, 2016; Soba, 2016). Self-avoidance (or isoneuronal repulsion) requires that dendritic branches emerging from the same neuron repel one another to prevent the entanglement of sibling dendrites. Similarly, tiling requires the dendrites of the same neuronal type avoid one another (Grueber and Sagasti, 2010), thereby allowing full-field coverage for complete input sampling but also protecting against input redundancy. By restricting sensory dendrites to non-overlapping fields, the tiling patterns of mechanosensory neurons can provide accurate locational information about a stimulus. The establishment of such distinct dendrite territories is thought to involve signals secreted by heterotypic neighbors or non-neuronal cells (Lefebvre et al., 2015; Parrish, 2016). Moreover, homophilic receptors, such as DSCAM in *Drosophila* and the clustered protocadherins (Pcdhs) in vertebrates, are necessary for the contact-mediated repulsion that allows dendrites to fill their target territories evenly, without intersection of sibling dendrites from the same neuron (Lefebvre et al., 2012; Figure 2E). Together, self-avoidance and tiling cooperatively ensure efficient and non-overlapping coverage of the receptive fields.

## Extrinsic Factors From Various Cells Impinge on Intrinsic Factors at Different Developmental Stages

Studies on *C. elegans*, *Drosophila*, Xenopus, and rodents suggest that dendritic morphogenesis proceeds in stages. In the initial targeting stage, primary dendrites extend away from the cell body or axon/dendrite shafts into appropriate target fields, where they may encounter the axon terminals of presynaptic partners. In the dendrite elaboration stage, highly dynamic cytoskeleton rearrangements and plasma membrane expansion are required for branching, growth and retraction of dendrites. As dendrites approach an appropriate level of coverage, self-avoidance and tiling mechanisms become major influences to prevent dendritic receptive fields from overlapping with neighbors. Dendrite growth is therefore restrained and stabilized as the dendrite arbors approach their proper borders. Lastly, during the dendrite remodeling stage, dendritic pruning can occur before synapse formation. Many secreted factors and receptors have been identified as regulators of these dendritic developmental stages (Table 1).

**TABLE 1:** Extrinsic factors regulate dendritic morphogenesis at different stages.

Extrinsic factor/receptor	Signal source	Effectors	System	Reference
**Initiation of dendritic outgrowth (Initiation stage)**				
Wnt (LIN-44)/Frizzled (LIN-17)	Posterior cell		*C. elegans* PQR neuron	Kirszenblat et al., 2011
Dscam1/Dscam1	Afferents	Dock/Pak/Cdc42	Fly embryonic CNS	Kamiyama et al., 2015
Semaphorin 3A (Sema3A)/Neuropilin-1 (Nrp-1)			Mouse hippocampal neurons	Shelly et al., 2011
**Dendritic branching and growth (Elaborating stage)**				
SAX-7, MNR-1/DMA1	Epidermis	Rac-WRC-Arp2/3	*C. elegans* PVD neuron	Zou et al., 2018
?/BAI1 (aGPCR)		RhoA	Rodent hippocampal neuron	Duman et al., 2019
?/BAI3 (aGPCR)		ELMO1/Dock1	Mouse Purkinje neuron	Lanoue et al., 2013
AMIGO2			Mouse SACs and RBCs	Soto et al., 2019
Wnt5a/Drl (Ryk)	Epidermis	Trio/RhoA	Fly ventral abdomen neuron	Yasunaga et al., 2015
TGF-β (Activin)/Babo	Afferents	dSmad2	Fly optic lobe	Ting et al., 2014
TGF-β/TGF-βR		Smad4/CRMP2	Mouse hippocampal neuron Human iPSC-derived neuron	Nakashima et al., 2018
GDNF/GFRα		NCAM1	Hippocampal pyramidal neurons dentate gyrus neurons	Irala et al., 2016; Bonafina et al., 2019
TGF-β (maverick)/Ret	Epidermis		Fly larval da neuron	Hoyer et al., 2018
FGF/FGFR1/2/3			Mouse somatosensory cortex	Huang et al., 2017
Insulin/InR	Afferents	PI3K/Tor/SREBP	Fly optic lobe	Luo et al., 2020
HSPGs (Dally and Sdc)/Ptp69D	Epidermis		Fly larval C4da neuron	Poe et al., 2017
L1CAM			Human ES induced neuron (iN)	Patzke et al., 2016
Nrg167/Nrg180 (L1CAM)	Epidermis		Fly larval C4da neuron	Yang et al., 2019
Dscam2/Dscam2	Afferents		Fly optic lobe (lamina)	Kerwin et al., 2018
Dscam1			Fly motoneuron	Hutchinson et al., 2014
Nectin-1			mouse olfactory mitral cell	Fujiwara et al., 2015
Sema3A/Nrp1-PlexinA4		FARP2/Rac1 CRMP2 CRMP4	Mouse cortical pyramidal neuron mouse CA1 pyramidal neuron mouse CA1 pyramidal neuron	Danelon et al., 2020; Niisato et al., 2013; Niisato et al., 2012
**Axon-dendrite fasciculation**				
SAX-3 (Robo)/SAX-7 (L1-CAM)	Afferents		*C. elegans* PVD neuron	Chen et al., 2019
**Dendritic guidance and targeting (Targeting stage)**				
Sema-2a/2b/Sema-1a	Afferents		Fly olfactory glomeruli	Sweeney et al., 2011; Shen et al., 2017
Wnt5/Drl-Vang	Afferents		Fly olfactory glomeruli	Wu et al., 2014; Hing et al., 2020
Ten-a and Ten-m	Afferents		Fly olfactory glomeruli	Hong et al., 2012
Fish-lips (Fili)	Afferents		Fly olfactory glomeruli	Xie et al., 2019
Toll-6 and Toll-7	Afferents		Fly olfactory glomeruli	Ward et al., 2015
Capricious	Afferents		Fly olfactory glomeruli fly optic lobe	Hong et al., 2009; Shinza-Kameda et al., 2006
Dscam2 and Dscam4	Afferents		Fly optic lobe	Tadros et al., 2016
Sidekicks, Dscams, and Contactins	Afferents		Mouse RGC	Krishnaswamy et al., 2015; Yamagata and Sanes, 2008; Yamagata and Sanes, 2018
Semaphorins/plexins			Mouse RGC	Koropouli and Kolodkin, 2014; Matsuoka et al., 2011a,b; Sun et al., 2013
**Dendritic self-avoidance and tiling**				
Dscam1	Isoneurons		Fly larval da neuron	Hughes et al., 2007; Matthews et al., 2007; Matthews and Grueber, 2011; Soba et al., 2007
DSCAM			Mouse RGC	Fuerst et al., 2009
Pcdhs	Isoneurons		Mouse SACs and Purkinje cells	Lefebvre et al., 2012
Sema6A/PlexA2			Mouse SACs	Sun et al., 2013
Slit2/Robo2			Mouse Purkinje cells	Gibson et al., 2014
UNC-6 (Netrin)/UNC-40/DCC			*C. elegans* PVD neuron	Smith et al., 2012
**Dendritic pruning (Remodeling stage)**				
Neuroglian (Nrg)			Fly ddaC sensory neuron	Zhang et al., 2014; Kanamori et al., 2015
EphBs/Ephrin-B3		Grb4/Dock180/PAK Pick1/syntenin	Mouse hippocampal CA1 neuron	Xu and Henkemeyer, 2009; Xu et al., 2011

### Dendrite Initiation Targeting Stage

Using hippocampal neuronal cultures as a model, previous studies revealed that the induction of neuronal polarity and the generation of single axons and multiple dendrites from the cell body proceeds in a well-defined temporal sequence (Cheng and Poo, 2012). Compared to axonogenesis (Arikkath, 2012; Chen et al., 2017), much less is known about how dendrite initiation is specified *in vivo*. For pseudounipolar neurons, dendrites first branch out at specific positions on the axonal shaft to innervate a specific target area. The initial targeting of dendrites thus influences the types of inputs that the neuron can receive. Recent studies revealed that Wnt(LIN-44)/Frizzled(LIN-17) and the transmembrane repulsive receptor, Dscam1, respectively, specify dendrite initiation sites in *C. elegans* oxygen-sensing PQR neurons (Kirszenblat et al., 2011) and *Drosophila* embryonic CNS neurons (Kamiyama et al., 2015). In vertebrates, the class 3 secreted Semaphorin-3A (Sema3A) and its receptor neuropilin-1 is involved in dendrite initiation in hippocampal neurons (Shelly et al., 2011).

### Dendrite Elaboration Stage

In the elaboration stage, dendrites undergo numerous extension and branching events to reach or cover appropriate target regions. Both dendritic extension and branching require substantial plasma membrane expansion and cytoskeletal reorganization (Menon and Gupton, 2018). Plasma membrane expansion in dendrites is fueled by membrane material transport *via* exocytosis and lipogenesis machinery (Peng et al., 2015; Meltzer et al., 2017; Ziegler et al., 2017; Urbina et al., 2018). Local and directed reorganization of the actin-cytoskeleton is also essential for dendritic extension and branching; the loss of cytoskeletal regulators generally leads to drastic alterations of dendritic structures (Coles and Bradke, 2015; Kapitein and Hoogenraad, 2015). In dendritic filopodia, linear and branched actin remodeling are thought to be tightly regulated by the Ena/VASP and WRC (WAVE Regulatory Complex) proteins, respectively. Furthermore, a recent study revealed that the Arp2/3 (actin-related protein 2/3) complex, under the control of the WAVE protein, serves as the major actin nucleator for branching initiation (Stürner et al., 2019).

### Dendrite Remodeling Stage

The remodeling or pruning process removes exuberant and excessive dendritic arbors as the nervous system matures (Riccomagno and Kolodkin, 2015). During *Drosophila* metamorphosis, dramatic remodeling/pruning of dendrites occurs in response to hormonal signaling by ecdysone. For instance, larval class IV dendritic arborization (C4da) neurons eliminate of all their dendritic branches, without affecting axons, before engaging the adult regrowth program (Kuo et al., 2005; Williams and Truman, 2005). In both insect and mammalian neurons, local activation of caspases is required for the elimination of dendritic branches and spines (Williams et al., 2006; Ertürk et al., 2014). The L1-type cell adhesion molecule, Neuroglian (Nrg), inhibits dendrite pruning in *Drosophila* ddaC sensory neurons, which depends on Rab5-dependent endocytosis-mediated degradation of surface Nrg (Zhang et al., 2014; Kanamori et al., 2015). While Nrg functions only in dendrites, the ephrin receptor, EphB3, has been implicated in both axon and dendrite pruning (Xu and Henkemeyer, 2009; Xu et al., 2011). Sema3A also regulates dendritic remodeling in an activity-dependent fashion in cultured hippocampal neurons *in vitro* (Cheadle and Biederer, 2014).

## Extrinsic Factors Influence Intrinsic Process *via* Local Actin-Cytoskeleton and Global Transcriptional Regulators

Dendritic morphogenesis depends on local modulation of cytoskeletal machinery and plasma membrane addition/expansion, which are crucial for dendrite extension and branching. Extracellular factors acting on cognate receptors are known to modulate these processes directly or indirectly to affect dendritic morphogenesis. Recent studies have identified two major mechanisms by which extrinsic factors drive dendritic morphogenesis: local modulation of adhesion or cytoskeletal components and global transcriptional regulation of key dendritic growth components.

### Transcription-Independent Mechanism

**FIGURE 3:**
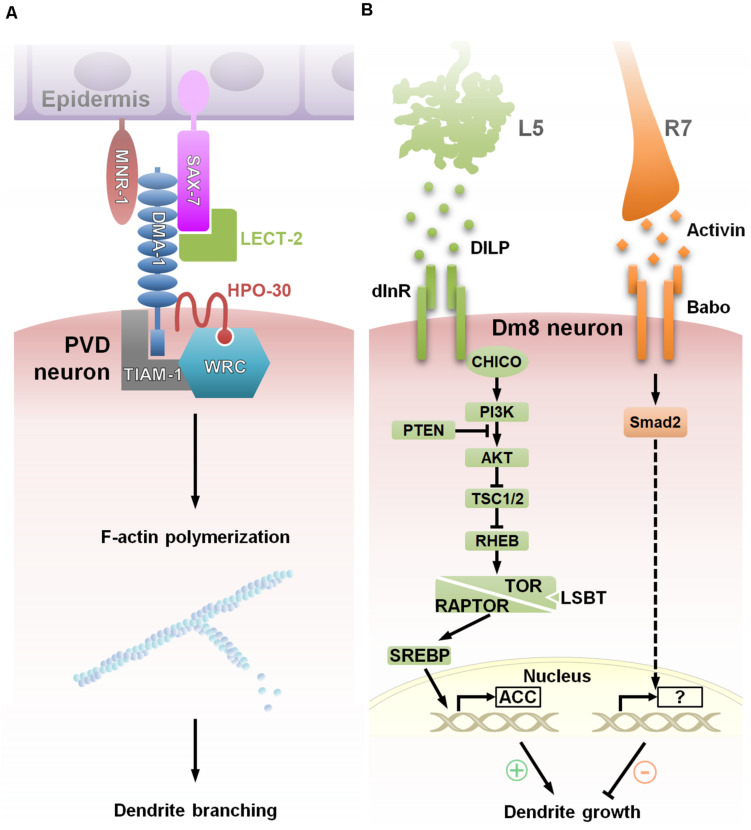
Extracellular factors govern dendritic morphogenesis *via* convergent signaling. **(A)** Model showing a multicomponent receptor-ligand complex regulating *C. elegans* PVD dendrite arborization. During the initiation of tertiary and quaternary PVD dendrite branches, membrane-associated protein, SAX-7/L1CAM, is expressed in a striped pattern in the underlying epidermis that correlates with the positions of dendrite branches. Surface expression of DMA-1 of PVD neuron receives extracellular signals *via* interactions with epidermal SAX-7/L1CAM and MNR-1, and the soluble ligand LECT-2 to function with HPO-30 and downstream effectors, TIAM-1 and the WRC (WAVE Regulatory Complex), that promote F-actin assembly, thereby resulting in dendritic branching at precise localization (Zou et al., 2018). **(B)** Two afferent-derived factors, Activin and DILP2, regulate Dm8 dendritic field size antagonistically. During the early pupal stage, insulin-like protein DILP2 derived locally from L5 neurons activates Insulin receptor (InR) and its canonical PI3K/AKT/TOR signaling pathway in Dm8 dendrites. Subsequent SREBP activation induces lipogenesis and stimulates Dm8 dendrite expansion. In the late pupal stage, InR expression declines, followed by the expression of Activin, which is derived from R7s. Activin acts on its receptor, Baboon, in Dm8 dendrites to restrict the expansion of the dendritic field. This temporal antagonistic regulation is accomplished by multiple afferent-derived morphogens and contributes to the robust and stereotyped control of Dm8 dendritic tree size (Luo et al., 2020).

Dendritogenesis largely depends on modulation of the actin and microtubule cytoskeleton. Live-imaging analysis shows that clusters of dynamic F-actin called “actin blobs” are recruited at branch initiation sites along dendritic shafts in *Drosophila* C4da neurons (Nithianandam and Chien, 2018), suggesting that at such branch initiation sites, actin-associated complexes facilitate dendritic branching. Many cell surface receptors and adhesion molecules associate directly with cytoskeletal machinery, thereby providing a means of directly translating environmental signals to local dendritic morphogenesis. One of the major convergence points is the WAVE regulatory complex (WRC), which binds and activates the Arp2/3 complex to drive actin polymerization at distinct membrane sites. WRC associates with diverse cell surface receptors, such as protocadherins, ROBOs and netrin receptors, in order to regulate dendritic morphogenesis (Chen et al., 2014). The most thoroughly examined example of this process comes from worm PVD neurons, in which the dendrite branching receptor, DMA1, and the claudin protein, HPO-30, form a signal-sensing complex with the RacGEF, TIAM-1, and WRC. In response to the epidermis-derived co-ligand complex, SAX-7/MNR-1/LECT-2, this system locally activates the Rac-WRC-Arp2/3 signaling pathway to promote F-actin assembly, which drives high-order dendritic branching (Zou et al., 2018; Figure 3A).

Sema3A, a secreted semaphorin highly expressed in cortical plate, patterns both dendrites and axons of cortical pyramidal neurons during development. Previous studies have shown that Sema3A signals through its receptor Neuropilin-1/PlexinAs to promote dendritic growth and branching *in vitro* and *in vivo* (Fenstermaker et al., 2004; Tran et al., 2009; Mlechkovich et al., 2014; Yamashita et al., 2016; Danelon et al., 2020). In this signaling complex, PlexinAs serve as signal-transducing subunits to bridge the extrinsic factor Sema3A and their downstream effectors that regulate cytoskeleton reorganization (see review Goshima et al., 2016). Tran and colleagues first demonstrated that Plexin-A4’s KRK motif which associate with the RhoGEF FARP2 is specifically required for dendritic branching but not growth cone collapse *in vitro* (Mlechkovich et al., 2014). Recently, they generated a *Plexin-A4^KRK–AAA^* knock-in mice and showed that activated Sema3A signaling initiates a novel Sema3A-Neuropilin-1/Plexin-A4/FARP2/Rac1 signaling pathway to mediate dendrite morphogenesis of layer-5 cortical neurons *in vitro* and *in vivo* (Danelon et al., 2020). Another downstream signaling pathway for Sema3A is the collapsin response mediator protein (CRMP) family, which is also linked to cytoskeletal modulation. CRMP2 appears to promote hippocampal pyramidal neuron apical dendrite branching (Niisato et al., 2013). In contrast, CRMP4 might be involved in pruning apical dendrites of olfactory mitral cells, as CRMP4 knockout mice have enhanced growth of mitral cell dendrites (Tsutiya et al., 2016).

Wnts are secreted glycoproteins that engage diverse signaling pathways on regulating different aspects of neuronal development. In contrast to its transcriptional role in neurogenesis and differentiation *via* the β-catenin-dependent pathway, Wnts function as instructive extrinsic signals and provide spatial information for regulating of F-actin assembly in axon/dendrite morphogenesis (He et al., 2018). In adult *Drosophila*, the boundary of the dendritic field in the ventral abdomen is controlled by repulsive Wnt signals from the underlying epidermal tissues. Wnt5a-Drl (Ryk in mammalian) interactions act through Trio, a Rho GTPase exchange factor to promote dendritic termination through the activation of RhoA, a regulator of actin-cytoskeletal dynamics (Yasunaga et al., 2015). Loss of Ryk, a non-canonical Wnt receptor, in mouse hippocampal and cortical neurons promotes dendrite growth and branching *in vitro*, whereas overexpression of wild type Ryk restricts these processes (Clark et al., 2014; Lanoue et al., 2017). Human patients of Williams syndrome, a genetic neurodevelopmental disorder, identified a mutation in a Wnt receptor, the *frizzled9* gene. It has been shown the downregulation of Wnt signaling increased total dendrite length in mutant neurons generated from patient-derived induced pluripotent stem cells (iPSCs) (Chailangkarn et al., 2016). These observations highlight an evolutionary conserved role of Wnt signaling in dendritic patterning.

The *Drosophila* embryonic aCC motoneuron serves as an especially illustrative example of how dendritogenesis sites are specified by coupling homophilic interactions to actin-cytoskeleton remodeling. *Drosophila* embryonic aCC motoneurons initiate dendritogenesis at sites of contact with the axons of MP1 neurons. In the aCC neuron, Dscam1-mediated homophilic interactions act *via* the Dock adaptor protein to localize the Cdc42 effector, Pak1, to the dendrite initiation site, thereby spatially restricting cytoskeletal remodeling (Kamiyama et al., 2015).

In another example from *Drosophila*, the attachment of dendrites to the extracellular matrix (ECM) confine da neuron dendrites to a 2D space, facilitating dendritic avoidance and tiling. The dendrite-ECM adhesion is mediated by interactions between dendritic integrins and epidermis-secreted laminins (Han et al., 2012). The semaphorin ligand, Sema-2b, is secreted by the epidermis and acts on the neuronal PlexB receptor to promote dendrite-ECM attachment (Meltzer et al., 2016). The Sema-2b/PlexB complex physically associates (and genetically interacts) with Mys, a β-subunit of integrin, and its downstream effectors, the TOR2 (target of rapamycin) complex and Tricornered (Trc) kinase. How Sema-2b/PlexB complexes activate Trc and/or modulate integrin activity to promote dendrite-ECM adhesion requires further investigation.

Contact-mediated extrinsic cues, such as intercellular recognition, establish dendritic patterning during development (Prigge and Kay, 2018). Adhesive molecules that regulate cell-cell recognition can lead to generation of a repellent signal or an adhesive interaction for establishing synaptic partnership (Sanes and Zipursky, 2020). Examples of trans-cellular binding-mediated adhesion are the Sidekicks and Teneurins, which function in specific laminar targeting of a subset of RGC dendrites in the vertebrate IPL (Figure 2A, Yamagata and Sanes, 2008, 2018; Krishnaswamy et al., 2015) and instruct dendritic targeting in *Drosophila* (Figure 2C; Hong et al., 2012), respectively. In both systems, either loss-of-function or gain-of-function result in the impairment of dendritic targeting. For repulsive interaction, the semaphorins and plexin signaling receptors are known as repellent signals for their roles in setting up laminar and cellular specificity. In the mouse retina, the transmembrane protein Sema6A and its receptors PlexinA2 or A4 are localized in specific sublaminae of the IPL. Loss of Sema6A severely disorganized lamina-specific arborization of RGCs and amacrine cells (Figure 2B; Matsuoka et al., 2011a,b; Sun et al., 2013). Similarly, loss of repellent effects of semaphorins also results in dendritic mistargeting in *Drosophila* olfactory system (Figure 2D; Shen et al., 2017). Recently, a study found that membrane leucine-rich repeat family member Fish-lips (Fili) acts as a non-homotypic repellent in ORNs signals to PNs, and in PNs signals to ORNs, to prevent invasion of neurites into inappropriate target region. Yet, the Fili receptor for this phenomenon is not known at this moment (Xie et al., 2019).

### Transcription-Dependent Mechanism

Transcriptional control, especially of membrane synthesis and cytoskeletal components/regulators, has emerged as a major mechanism for extrinsic factors to modulate global dendritogenesis. Growth of large and highly branched dendrites requires a continuous supply of membrane constituents, which is generated by *de novo* lipid synthesis. Sterol regulatory element binding protein (SREBP) is a key transcription factor for lipogenic gene expression, and silencing of SREBP was found to reduce dendrite branching and length in *Drosophila* da neurons (Meltzer et al., 2017; Ziegler et al., 2017). A recent study on *Drosophila* Dm8 dendritic development provides a link between extrinsic factors and SREBP-dependent transcription regulation (Luo et al., 2020). In response to the afferent-derived insulin-like peptide, Dilp2, Dm8s activate the canonical InR/PI3K/TOR1 pathway, which activates SREBP to promote dendritic growth (Figure 3B). Whether SREBP activity is regulated by insulin or other extrinsic factors in *Drosophila* da neurons remains unknown.

In contrast, Smad-mediated transcriptional control negatively regulates dendritic growth and branching. In mouse hippocampal neurons and human iPSC-derived neurons, activated Smad interacts with the transcriptional repressor, TG-interacting factor (TGIF), to silence the expression of the CRMP2, a cytoskeleton regulator involving in dendrite elongation (Nakashima et al., 2018). By antagonizing the growth-promoting effects of the InR/PI3K/TOR1 pathway, TGF-β/Activin signaling restricts dendritic arborization of Dm8 and Tm20 medulla neurons, also through Smad-mediated transcriptional regulation (Ting et al., 2014; Figure 3B). Activin derived from afferent R7s and R8s acts specifically on the Baboon receptors, respectively, expressed by Dm8 and Tm20 neurons to activate the Smad2 transcription factor. While Smad2 appears to affect dendritic termination frequency, the transcriptional targets of Smad2 in Dm8 and Tm20 neurons have yet to be identified.

## Combinatorial Extrinsic Factors Coordinate Dendritic Development

It has been suggested that combinatorial molecular codes is the common principle of brain wiring for overcoming limited numbers of molecules as compared to the complexity of the nervous systems. Unlike axon guidance which employs multiple guidance receptors (Richardson and Shen, 2019), less is known about the combinatorial codes of extrinsic factors in dendritogenesis. The dendrite morphogenesis of the worm PVD neurons employees a unique combinatorial coding strategy of multi-ligand-receptor assembly. Proper dendritic patterning, especially dendritic branching, is driven by a penta-partite ligand-receptor complex formed by two dendritic receptors (DMA-1 and HPO-30), two epidermis transmembrane ligands (SAX-7 and MNR-1) and the muscle-secreted ligand LECT-2 (Zou et al., 2018; Figure 3A). In *Drosophila* class III da neurons, the Dscam1-mediated self-repulsive mechanism works cooperatively with the Netrin/frazzled guidance cue to guide sensory dendrites to their targets while uniformly filling the target field (Matthews and Grueber, 2011). Below we discuss a number of examples of multiple signaling pathways converging intracellularly to generate unique dendritic patterns.

### Combinatorial Codes for Glomerulus Targeting

Recent studies have focused on the cues that mediate early dendritic targeting of olfactory projection neurons (PNs) to glomeruli of the *Drosophila* antennal lobe (AL). One such study revealed that the transmembrane cell surface receptor, Semaphorin-1a (Sema-1a), displays a graded expression pattern in the AL, with the highest protein level in PN dendrites at the dorsolateral corner (Komiyama et al., 2007). Based on loss-of-function experiments in several PN types, Sema-1a was initially proposed to instruct coarse PN dendritic targeting along the dorsolateral-ventromedial (DL-VM) axis by its action as a receptor for an opposing gradient of repulsive guidance cues (secreted ligands, Sema-2a/2b) from axons of degenerating larval olfactory receptor neurons (ORNs) (Sweeney et al., 2011). However, a more comprehensive analysis of *Sema-1a* mutants in many PN types suggested that Sema-1a functions locally to prevent PN dendrites from mis-targeting to select AL regions. The dendritic mis-targeting in multiple *Sema-1a* mutant PN types was inconsistent with the predictions of the original semaphorin gradient model (Figure 2C; Shen et al., 2017), challenging the idea that Sema-1a globally controls PN dendritic targeting along the DL-VM axis of the AL. Since PNs precisely project dendrites to unique AL glomeruli in wild-type animals, these Sema-1a studies raise the possibility that combinatorial molecular codes incorporate Sema-1a to ensure the generation of discrete dendritic patterns among distinct PN types. Molecules with graded expression, other than Sema-1a, may then be responsible for globally directing PN dendritic targeting within the AL. An excellent candidate is the repulsive guidance cue Wnt5 which forms a DL-high to VM-low gradient that orients specific PN dendrites. Moreover, an ORN axon-derived transmembrane planar cell polarity (PCP) protein, Van Gogh (Vang), serves as a mediator of Wnt5 repulsion in the context of PN dendritic targeting. Interestingly, PN dendrites express different levels of Drl (a Wnt5 receptor) to antagonize the Wnt5-Vang repulsion and direct appropriate localization to glomerular positions (Wu et al., 2014; Hing et al., 2020). By utilizing combinatorial molecular codes both locally and globally, proper dendritic patterns can be established among distinct PN types, permitting appropriate synapse formation with partner ORNs to create an accurate olfactory map.

### Matching Pre/Post-synaptic Partners *via* Ig Superfamily Adhesive Code

An additional example of a known molecular combinatorial code is related to the Ig-containing adhesive receptor Dscams in fly visual laminar neurons (Zipursky and Grueber, 2013; He et al., 2014; Lah et al., 2014; Tadros et al., 2016). In each photoreceptor synapse, there is a tetrad of postsynaptic elements that invariably incorporates paired dendrites of laminar neurons, L1 and L2. Reportedly, L1 and L2 cells express different sets of Dscam1 and Dscam2 proteins (L1 expresses the Dscam2B isoform; L2 express Dscam2A) (Lah et al., 2014). Loss of either Dscam1 or Dscam2 produces mild pairing defects, and both Dscam1 and Dscam2 are required for correct postsynaptic pairing with a photoreceptor in single cartridge. When two dendrites from the same cell encounter each other, Dscam1 and Dscam2 stimulate homophilic repulsion to promote self-avoidance, preventing L1/L1 or L2/L2 pairs from incorporating into the same tetrad (Millard et al., 2010). These findings suggest that pairing of L1 and L2 may require other adhesive molecules, and together, they illustrate an extrinsic molecular combinatorial code that ensures proper dendritic morphogenesis.

### Robust Dendrite Size Control by Two Afferent-Derived Secreted Factors

The multi-ligand combinatorial control of dendritic patterning is exemplified by a recent study characterizing dendritic size control of *Drosophila* Dm8 amacrine neurons (Luo et al., 2020), which ramify large dendritic arborizations to receive ∼14 inputs from R7 neurons (Gao et al., 2008). An earlier study showed that R7s secrete the TGF-β superfamily ligand, Activin, to restrict expansion of the Dm8 dendritic field (Ting et al., 2014). The work by Luo et al. then revealed a counteracting mechanism, in which the insulin ligand/receptor system promotes Dm8 dendritic arbor growth. Upon removal of L5 lamina neuron-derived Insulin-like Peptide 2 (DILP2) or disruption of insulin/Tor signaling in Dm8s, the dendritic arbors of Dm8 neurons are reduced and synapse with fewer photoreceptors (Figure 3B). A single-cell experiment further revealed that insulin signaling is under spatiotemporal control in Dm8s. As such, Dm8 neurons exhibit transient insulin receptor expression at early pupal stages, a time at which the cells have just begun to expand their dendritic arbors. Thus, Dm8s appear to receive both positive (insulin) and negative (Activin) signals to regulate their dendritic field size. Despite the antagonistic actions of Activin and DILP2, both are derived from afferents transmitting in a circuit-specific manner and acting on Dm8 dendrites at close range. Both morphogens are also generated by other adjacent afferents (DILP2 from L3 neurons and Activin from R7s), however, morphogens produced by those more distant sources are not necessary for normal Dm8 dendrite development. These observations suggest a precise spatial regulation in this context and support the general idea that afferent-derived cues tend to function at short range during distinct developmental stages. Observations from genetic interaction experiments further suggested that Activin signaling acts in parallel with insulin signaling through TOR and SREBP to control Dm8 dendrite elaboration. Interestingly, removing both signaling events causes Dm8 neurons to exhibit a normal average but highly variable dendritic field size, suggesting the antagonistic regulation by multiple afferent-derived morphogens is required for robust control of Dm8 dendritic tree size (Luo et al., 2020).

Previous theoretical studies using modeling and simulations have shown that the dendritic kinetic parameters, such as branching and terminating frequency, can determine the size and complexity of a dendritic tree (Cuntz et al., 2010; van Elburg, 2011; Lin et al., 2018; Luo et al., 2020); high branching and low terminating frequencies favor dendritic growth and result in large and complex dendritic trees, and vice versa. Interestingly, the robustness of dendritic tree sizes can be correlated with the ratio of terminating and branching frequencies. As such, high branching frequency that approximates the terminating frequency produces large but highly variable dendritic trees. Monte Carlo simulations further suggest the elaboration of both large and consistent dendritic trees can be achieved by temporal regulation of these two parameters. For example, large consistent dendritic trees can be generated by favoring growth in the early stage and increasing terminating frequency at later stages of development. In the Dm8 system, growth-promoting insulin signaling is normally restricted to early developmental stages, and ectopically extending the expression of insulin receptors resulted in highly variable dendritic field sizes (Luo et al., 2020), supporting the temporal regulation model.

## Conclusion and Future Challenges

To form stereotypic dendritic arbors, neurons endowed with specific intrinsic properties, such as cell-type-specific transcription programs, must respond appropriately to extrinsic factors (environmental cues) to properly execute dendritic morphogenesis during development. Studies over the past decades have uncovered a broad range of extrinsic factors, including morphogens, growth factors and adhesive receptors, that are provided by afferents or surrounding cells to affect various aspects of dendritic growth and patterning. These extrinsic factors act on cognate receptors to regulate global transcription or modulate local cytoskeletal organization and adhesion, in order to size, shape, and localize dendrites. Many of the identified extrinsic factors, receptors and downstream effectors are utilized in shaping dendrites across different systems. However, depending on the specific neural architectures, such as glomerular or layer-column structures (and hence the patterns of extrinsic factor expression), the effects of the machinery on final dendritic patterning are translated into glomerular targeting, layer-specific targeting, or receptive field establishment that meet the needs of the specific neurons.

One major challenge that lies ahead is to decipher the logic and potential hierarchy of combinatorial codes of intrinsic and extrinsic factors. Open questions remain as to how multiple extrinsic factors coordinate in a spatiotemporal fashion to shape dendrites and how combinations of cell-specific intrinsic factors and environmental cues give rise to cell-specific dendritic patterns and connectivity. Recent advances in single-cell transcriptomics might provide a means to identify and decipher the combinatorial molecular codes that generate complex and cell-type-specific dendritic patterns (Li et al., 2017; Kurmangaliyev et al., 2019; Davis et al., 2020). Stage-dependent gene expression, as revealed by developmental single-cell transcriptomics, has hinted at the importance of temporal regulation of extrinsic factors and receptors (Jain et al., 2020; Kurmangaliyev et al., 2020; Özel et al., 2020). One study suggested that the temporal regulation of receptors and antagonistic regulation are required for robust control of dendritic sizes (Luo et al., 2020), while other mechanisms of spatiotemporal regulation and combinatorial codes are being uncovered. However, it would be difficult to derive a comprehensive understanding of these processes without direct examination of the dynamic processes of dendritic patterning in developing brains (Sheng et al., 2018).

Dendritic morphological defects have been found in patients with various neuropsychiatric disorders of developmental origin. While understanding how dendritic patterning defects cause connectivity and functional deficits is an important goal in its own right, such studies may also reveal how crucial aspects of dendritic development are constrained by functional requirements. Current connectome studies provide critical reference maps, and future advances might allow for complete analysis of synaptic circuits in mutant brains. Nonetheless, recent studies using light microscopic techniques, including the activity-dependent GRASP (GFP reconstitution across synaptic partners) method, have already begun to uncover connectivity abnormalities associated with dendritic patterning defects, and functional studies using electrophysiology or functional imaging will likely follow suit. “Form ever follows functions,” the dictum of the famous architect, Louis Sullivan, provides a useful perspective for studying dendritic morphogenesis. By linking genes to connectivity and to functions, studies of dendritic development in the brain might reveal the logic of the greatest architect, nature.

## Author Contributions

T-YL drafted the original manuscript. C-HL conceived the scope of the review and did the supervision. H-HY, C-PH, and P-JC contribute to the writing of this review and the generation of figures. All authors contributed to the article and approved the submitted version.

## Conflict of Interest

The authors declare that the research was conducted in the absence of any commercial or financial relationships that could be construed as a potential conflict of interest.
